# Aggression from Patients or Next of Kin and Exposure to Bullying Behaviors: A Conglomerate Experience?

**DOI:** 10.1155/2017/1502854

**Published:** 2017-02-08

**Authors:** Iselin Reknes, Guy Notelaers, Nils Magerøy, Ståle Pallesen, Bjørn Bjorvatn, Bente Elisabeth Moen, Ståle Einarsen

**Affiliations:** ^1^Department of Psychosocial Science, University of Bergen, Bergen, Norway; ^2^Department of Occupational Medicine, Haukeland University Hospital, Bergen, Norway; ^3^Norwegian Competence Center for Sleep Disorders, Haukeland University Hospital, Bergen, Norway; ^4^Department of Global Public Health and Primary Care, University of Bergen, Bergen, Norway

## Abstract

Although workplace violence and aggression have been identified as important stressors in the nursing profession, studies simultaneously comparing patient-initiated aggression and exposure to bullying behaviors at work are rather scarce. The aim of this study was to compare aggression from patients or next of kin and exposure to bullying behaviors in terms of prevalence, health-related quality of life outcomes, and potential overlap in those targeted. In the period of 2008-2009, data were collected among 2059 members of the Norwegian Nurses Organization. Latent class (LC) analysis and a multivariate analysis of variance (MANOVA) were used to investigate the proposed relationships. The results showed that aggression from patients or next of kin and exposure to bullying behaviors were perceived as separate and independent stressors. Although aggression from patients or next of kin was more frequent than workplace bullying, the latter was the only significant stressor related to health-related quality of life in terms of reduced mental health functioning. Although being a rather infrequent experience, exposure to bullying behaviors seems to have more severe health-related outcomes for nurses than aggression from patients or next of kin. Hence, the results of the study strengthen previous findings and suggest that managers must aim to maintain a positive psychosocial work environment with zero-tolerance for bullying.

## 1. Introduction

Nurses are part of a professional group known to be at risk of workplace violence and aggression [1], which includes risks of being exposed to physical assaults, threat of assaults, and verbal abuse [2]. The negative behavior may include various perpetrators and may take many forms [3], yet mainly being either consumer-related violence (i.e., violence and aggression by clients and/or patients against staff) or relationship violence (i.e., exposure to harassment and bullying at work by one's colleagues or superiors) [4]. Although nurses may have close working relationships with both patients and colleagues, violence from these groups may happen in isolation and therefore be evaluated by nurses as different and separate phenomena when experienced. Yet, these types of violence may also occur simultaneously or be sequentially linked to each other and thus be experienced as more or less conglomerate stressors. Some nurses may also be vulnerable to both. In line with this, previous research has stated a need for more research including the outcomes of both insider and outsider initiated violence (e.g., [5, 6]). Even though some studies have addressed this call [6–8], studies simultaneously comparing health-related outcomes of aggression from patients or next of kin and exposure to bullying behaviors are still warranted (for an exception see [9]). Hence, the aim of the present study is to investigate the occurrence and overlap between aggression from patients or next of kin and exposure to bullying behaviors and to determine their relative relationship with health-related outcomes and well-being.

## 2. Background

### 2.1. Aggression and Bullying at Work

Aggression has been defined as goal-directed behavior with intent to harm or injure another person or object [10]. Aggressive behaviors can be of a sporadic character, also describing one-time occurrences, carried out by various types of perpetrators [3, 11–13]. Yet, aggression may also become a constant threat even if the actual sources tend to vary. Workplace bullying on the other hand is characterized as a process of negative behaviors including harassing, offending, or socially excluding someone or negatively affecting someone's work. The behaviors occur repeatedly and regularly over a period of time, often involving a power imbalance between the target and the perpetrator, where the target tends to end up in an inferior position (see [14]). Hence, workplace bullying is a process where the target is exposed to negative behaviors over time often by the same perpetrator(s) who are among one's peers and/or superiors [15].

A review of the prevalence of patient-initiated violence showed that the reported proportion of verbal abuse varied from 22% to 90%, threat of violence varied from 12% to 64%, and physical violence varied from 2% to 32% [16]. On the other hand, approximately 3% to 15% of the European workforce may have experienced serious or occasional bullying at work [17], hence being a less prevalent problem. Some studies indicate, however, that employees in the social and health sector are at a higher risk of being subjected to bullying behaviors from colleagues or superiors than employees in general (see [17]). Illustrative of this, the prevalence of bullying at work among nurses varies from 4% [18], via 21% [19], 34% [9], 44% [20], to 86% [21].

In general, however, studies tend to show that aggression from patients or consumers is more prevalent than aggression from colleagues or superiors [2, 5, 16, 22]. Previous studies comparing the prevalence of aggression from patients and bullying from colleagues in the nursing profession have, however, shown quite similar prevalence of the two stressors: 36% versus 32% [23], 29% versus 26% [24], and 30% versus 34% [9]. Hence, although previous research shows that aggression from patients is being more prevalent than aggression from colleagues among nurses (e.g., [25]), the picture is blurred when it comes to patient- or next of kin-initiated aggression versus workplace bullying.

### 2.2. Health Outcomes of Aggression and Bullying at Work

Aggression and bullying may affect the nurses in several ways. Differences in how nurses perceive aggressive and bullying behaviors may differ according to several factors: how they perceive the situation, who the perpetrators are, how they define what they are exposed to, and whether they believe it to be part of their job (see [26, 27]). When perceived as a stressor, however, any exposure to violent or aggressive episodes from patients or colleagues may likely result in negative outcomes [28]. Being exposed to outsider violence (i.e., from customers, clients, patients, or others) has been associated with both physical and psychological health problems for those targeted [29]. For instance, in a systematic review of nonsomatic effects of patient assaults [30], the predominant responses were anger, fear or anxiety, posttraumatic stress disorder symptoms, guilt, self-blame, and shame. Also, nurses seem to react similarly across different cultures and settings [30]. Furthermore, strong relationships between bullying from colleagues and health outcomes, like burnout [31], psychological distress [32], and depression [33], are documented among nurses.

Being exposed to stressful events at work is likely to increase the target's level of cognitive activation, which according to Harvey [34] can be described as worrying or repetitive thoughts, triggering autonomic arousal and emotional stress. Previous research has shown that worrying is related to somatic health problems [35]. This corresponds with the Cognitive Activation Theory of Stress (CATS; [36]), which states that prolonged cognitive and physiological activation, as a result of being exposed to a stressor evaluated as threatening over time, will increase the risk of impaired health when the target feels unable to cope with the situation. The duration of exposure seems, however, to affect the target's reactions to the stressor. Hence, as aggression typically is of a more sporadic character than workplace bullying [11, 12], which is defined as repeated and sustained exposure, it is likely that the latter will have a more severe impact on the targets' health due to the assumptions in CATS. In line with this, worrying has been shown to act as a mediator on the relationship between bullying and sleep quality [37].

An additional explanation of the proposed difference in health-related outcomes of aggression from patients or next of kin as compared to exposure to bullying behaviors may be the tendency to put people in the in-group according to the level of identification [38]. People similar to oneself can be labeled as members of the in-group while people different from oneself are labeled as members of the out-group [39]. Nurses are therefore likely to see their colleagues as a part of their in-group of whom they compare themselves to. Being excluded from social events or from the fellowship at work (i.e., from the in-group) could make the targets nervous and anxious, due to the lack of a sense of belonging [40]. In a hospital setting, patients, on the other hand, usually stay for a short period of time, and the continuous replacement of patients may contribute to the evaluation of patients as members of the out-group with less impact on the nurses' health and well-being. Nurses may also perceive bullying behaviors from colleagues as something different and more harmful than aggression from patients or next of kin, just because the latter problem is more prevalent and in many cases may be considered as “part of the job” by nurses (e.g., [41]).

To our knowledge, a comparison of health-related outcomes of aggression from patients or next of kin and workplace bullying has only been addressed in one previous study, conducted among Australian nurses and midwives [9]. In Demir and Rodwell's [9] study, the results showed that both bullying from colleagues and aggression, in the form of verbal sexual harassment from patients and their family members, were linked to higher levels of psychological distress. This result was explained according to self-attribution processes (see also [42]), where nurses blame themselves for the occurrence of these stressors, leading to psychological stress. However, in a study by Farrell and Shafiei [23], the results showed that nurses were less worried by patient-initiated aggression compared to bullying from colleagues, because the latter made them feel less safe and confident at work. Hence, it may be that bullying from colleagues generates other consequences for those affected compared to aggression from patients or next of kin.

### 2.3. Aims of the Present Study

Previous studies have shown that outsider aggression has less impact on the employees' work experience, because the perpetrators are not members of the organization, and thereby the employees may be less likely to blame the organization for these negative encounters (see [6, 7]). Hence, exposure to bullying behaviors may have more negative outcomes for those targeted despite the presumably lower prevalence compared to outsider aggression. Following this, we want to investigate the relationship between aggression from patients or next of kin and exposure to bullying behaviors at work in terms of whether these stressors appear separately or overlap in terms of targets, hence being perceived as a conglomerate experience. In this study, we are also interested in how prevalent these two stressors are. Based on previous research, we would expect aggression from patients or next of kin to be more commonly experienced than exposure to bullying behaviors. Furthermore, based on previous research and the stressors' difference in nature of sustained exposure (see also CATS: [36]), we would expect exposure to bullying behaviors to have more severe health-related outcomes for those targeted compared to aggression from patients or next of kin. The following hypotheses will be investigated.

**Hypothesis 1 .**

Among nurses, aggression from patients or next of kin is more commonly experienced than workplace bullying.

**Hypothesis 2 .**

Exposure to bullying behaviors is more strongly related to negative health-related quality of life outcomes than aggression from patients or next of kin.

## 3. Methods

### 3.1. Design

The study is based on cross-sectional data from the Survey of Shift work, Sleep and Health (SUSSH), collected in 2008-2009 among members of the Norwegian Nurses Organization (NNO). A recommendation letter of the study from NNO, an information letter explaining the purpose of the study, a questionnaire, and a prepaid envelope for return were sent to all participants' home address.

### 3.2. Sample

An invitation to participate in the study was initially sent to 6000 Norwegian nurses. However, due to a number of wrong addresses, the invitation was received by 5400 nurses, of whom 2059 responded (38.1% response rate). The sample consisted of 1857 (90.2%) women and the mean age was 33.1 years (SD = 8.17), ranging from 21 to 63 years.

### 3.3. Measures

Nine items from the Negative Acts Questionnaire (NAQ; [43]) were used to measure exposure to bullying behaviors at work. This short NAQ instrument [44] measures negative behaviors which may be perceived as bullying if systematically repeated over time, that is, (1)* “someone withholding information which affects your performance,”* (2)* “repeated reminders of your errors or mistakes,”* (3)* “persistent criticism of your work or work-effort,”* (4)* “spreading of gossip and rumors about you,”* (5)* “having insulting or offensive remarks made about your person, attitudes or your private life,”* (6)* “being ignored or excluded,”* (7)* “being ignored or facing a hostile reaction when you approach,”* (8)* “practical jokes carried out by people you do not get along with,”* and (9)* “being shouted at or being the target of spontaneous anger.” *Respondents were asked how often, during the last six months, they had been exposed to such negative behaviors at their workplace with responses scored on a 5-point scale from 1 =* “Never”* to 5 =* “Daily*.*”* Cronbach's alpha for this scale was .75.

Aggression from patients or next of kin was measured with three items asking how often, during the last 6 months, the respondents had been exposed to (1)* “difficult patients or next of kin,”* (2)* “verbal assaults or spontaneous tantrums from patients or next of kin,”* and (3)* “physical assaults or threats of such assaults from patients or next of kin.”* Responses were given on a 5-point scale from 1 =* “Never”* to 5 =* “Daily*.*”* Cronbach's alpha for this scale was .81.

Health-related quality of life outcomes were measured with the SF-12v2. Two summary scores were used, one Mental Component Summary Score (MCS) and one Physical Component Summary Score (PCS), which have been calculated on an algorithm from the user's manual for the SF-12v2® health survey (range: 0–100) [45]. High scores on these component summary scores are positive in terms of good health-related quality of life.

Age and gender were included as control variables as these factors have been related to differences in exposure to violence at work [46].

### 3.4. Ethical Approval

The study was approved by the Regional Committee for Medical and Health Research Ethics, Health Region West (REK-Vest). Moreover, participation was voluntary, and the respondents were allowed to resign from the study at any time. Also, they had to provide informed consent in written form before being included in the study.

### 3.5. Analytic Strategy

Following recent trends in the measurement of workplace bullying, and in particular the identification of targets, we used latent class (LC) analysis in the present study to determine the relationship between aggression from patients or next of kin and exposure to bullying behaviors. LC analysis is very suited because it is not dependent upon distributional assumptions [47, 48]. LC analysis [49–53] is a statistical method that systematically classifies respondents into mutually exclusive groups with respect to a given trait (e.g., exposure to workplace bullying) that is not directly observed (manifest). The classes are not directly observable; they are latent [54]. Furthermore, one empirically investigates whether the assumption about the relationship between the latent variable (e.g., experiencing bullying/aggression) and the frequencies of reported behaviors (e.g., negative social behaviors/aggressive behaviors) is acceptable [48]. LC analysis enables the researcher to identify mutually exclusive groups that adequately describe the dispersion of observations in the *n*-way contingency table of discrete variables (i.e., workplace bullying/aggression). The goal of LC analysis is to determine the smallest number of latent classes, sufficiently explaining (or accounting for) the associations observed between the manifest variables (the twelve items in our study) [53].

The starting point for a latent class model is homogeneity; that is, everybody resides in the same group. This model is a 1-latent class cluster (LCC) model. Instead of increasing the number of latent class clusters, however, the number of latent variables (factors) may be increased as well. Magidson and Vermunt [51] label this type of LC models as latent class factor (LCF) models because of the natural analogy to standard factor analysis. This would follow the assumption that aggression from patients or next of kin and exposure to bullying behaviors are different constructs for the nurses included in this study. Like with traditional confirmatory factor models, a priori knowledge about the relationship between items and latent variables is needed [55]. As in traditional reflexive measurement models, the discrete latent variable must adequately explain the initial relationship between the indicators.

For the examination of the relationships between aggression from patients or next of kin and exposure to bullying behaviors, the software package Latent Gold 5 was used [56]. Evaluating the fit of LC models is not straightforward. There are many indicators of fit and rules of thumb that should be taken into account. The Bayesian Information Criterion (BIC) is most often used, and among others McCutcheon [57] and Hagenaars [58] suggest accepting the model with the lowest BIC. However, for very sparse tables, it is likely that the squared log-likelihood (*L*^2^) will not follow a chi-squared (*χ*^2^) distribution. Therefore, Langeheine et al. [59] suggested a bootstrapping procedure that was implemented in Latent Gold. This procedure is used here because bullying and aggression items are highly skewed and many combinations of specific negative acts are uncommon, which results in very sparse tables. This bootstrapping procedure is used in two ways. Firstly, it is used to assess the overall fit of a table where *p* values equal to or higher than 0.05 indicate overall fit. Secondly, it is used to determine whether adding latent classes leads to an improvement of fit. Hence, the difference in −2LL with a certain difference in degrees of freedom is bootstrapped. A nonsignificant *p* value indicates that the model does not improve with an additional class, whereas a significant *p* value (<0.05) conversely indicates an improvement of fit. In addition to the BIC and the bootstrapping procedure, it is also important to inspect the bivariate residuals (BVR). The BVR show how much association between each pair of indicators remains, using the 1-cluster model as a reference. Ideally, the value should be lower than 3.84, being a value which corresponds to significant *χ*^2^ with 1 degree of freedom [60]. However, as *L*^2^ follows *χ*^2^ distribution, the BVR is also quite sensitive for large sizes. Therefore, we suggest using a more relative threshold, where the reduction of the BVR should be at least 90% [47]. Finally there are also more descriptive measures to assess fit. A much used approach is to inspect the proportion reduction in *L*^2^. Being a global fit measure, the proportion reduction of error (PRE) expresses how much *L*^2^ has been reduced, compared to the 1-cluster model, which serves as a baseline model. Furthermore, we also assessed how well classes were separated and inspected *R*^2^, entropy *R*^2^, and the total rate of classification errors due to adjacent erroneous classification.

After the measurement model was established, the latent class classifications were exported to a SPSS file [61]. A multivariate analysis of variance (MANOVA) was conducted in order to simultaneously compare health-related quality of life outcomes, measured by one physical health component summary score and one mental health component summary score (SF-12v2), of aggression from patients or next of kin and exposure to bullying behaviors. This was done in order to avoid a type I error inflation because of a multiple testing procedure that would arise from repeating independent analyses of variance (ANOVAs).

## 4. Results

### 4.1. Selection of Appropriate Measurement Model

Table 1 gives an overview of the different measurement models estimated with Latent Gold 5 and their respective fit measures. In particular, we evaluated fit with multiple measures that are the Bayesian Information Criterion (BIC) based upon the log-likelihood, the number of parameters in the statistical model (*N* par), the squared log-likelihood (*L*^2^), the proportion reduction of error, which approximates the explained variance of the model, the bootstrapped *p* value of the squared log-likelihood, and, finally, the percentage of adjacent classification errors.

As indicated above, establishing fit of a latent class model is complicated, and different measures must be assessed. Among the factor models with no error correlation, the factor model which distinguished 6 latent classes for workplace bullying and 5 latent classes for aggression from patients or next of kin had the lowest BIC. However, the bootstrap of *L*^2^ indicated that the model was significant. The bootstrapped –2LL difference test, which establishes whether it is statistically sound to add classes, yielded clearly that a 2-factor model, modelling 5 levels for each factor, was fitting less well. Indeed, distinguishing more latent classes like 6 classes for workplace bullying was associated with a nonsignificant bootstrapped −2LL difference test, which thus yielded deterioration of fit. The inspection of the bivariate residuals of the model with 5 levels for each factor showed that within the factors themselves the initial bivariate residuals were sufficiently explained. However, several bivariate relationships between aggression and bullying indicators were not explained for more than 90%, indicating a potential need for allowing cross-loadings instead of adding latent classes [62]. Hence, we allowed the first item of the bullying measure (i.e.,* someone withholding information which affects your performance)* to correlate (*r* < 0.20) with the first item of the aggression measure (i.e.,* difficult patients or next of kin)*. This model is depicted in the last row of Table 1.

Altogether the fit indices yielded that this 2-factor model, where each of the factors has 5 different (ordered) classes, respectively, was the most suited model. More specifically, the BIC was the lowest and, importantly, the *p* value of the *L*^2^ bootstrap procedure was nonsignificant (*p* = 0.05), which indicates that this model fitted the data well. We note that a single latent class factor model did not fit the data better than a 2-latent-class-factor model. Furthermore, the correlation between the factors was only 0.28, which indicates that aggression from patients or next of kin and exposure to bullying behaviors were only weakly related and thereby not reflecting the same concept. Hence, these stressors are seen as different construct in the eyes of the beholder, in our case nurses. All conditions mentioned above support the notion that these stressors can be regarded as two separate factors.

### 4.2. Meaning of the Latent Classes of Aggression and Workplace Bullying

Conditional probabilities allowed us to depict the precise meaning of the latent classes. Given that we used five response categories for each indicator, the table should have been at least sixty rows (twelve items with 5 response alternatives each) by 10 columns long (2 factors with 5 different classes each). We chose to simplify this table by portraying the conditional means in Table 2. These means are the average score to an item given the latent class membership (for a more detailed view, please consult the first author). Additionally, the size of the different classes for each factor is presented in percentages on the first row.

The exposure to bullying behaviors factor consisted of 5 latent classes. In the first class, hardly any exposure to bullying behaviors was reported, leaving us with the label of* “never bullied”* for this class. In the second class, there was a slightly higher frequency of bullying behaviors reported, although it was still low, and we decided to label this class* “hardly any bullying behaviors.” *In the third class, the average of the nine items increased further but was still below 2. Therefore, we suggest labeling this class* “rarely bullied.”* In the fourth class, average of exposure points to occasional reports of bullying behaviors, indicating that* “occasionally bullied” *is a suitable label for this class. Finally, in the fifth class, all averages were above 2, except for the sixth item (i.e.,* being ignored or excluded)*, indicating that the frequency of bullying behavior was highest in this class. Thus we suggest labeling this class* “frequently bullied*.*”* Meanwhile 50% were not exposed at all to any bullying behaviors at work, 6% were hardly exposed to bullying behaviors, 29% were rarely exposed to bullying behaviors, 13% were occasionally exposed, and, finally, 2% were more frequently exposed to bullying in the current sample (Table 2, first row).

With respect to aggressive behaviors from patients or next of kin, the latent class factor also consisted of 5 latent classes. In the first class, hardly any exposure was reported. This class was therefore labeled* “never exposed to aggression*.*”* In the second class, aggressive behavior was rarely reported expect for the first item where respondents on average claimed to have experienced it occasionally; hence, we labeled this class* “rarely exposed to aggression*.*”* In the third class, the average of both the first item (i.e.,* difficult patients or next of kin)* and the second item (i.e.,* verbal assaults or spontaneous tantrums from patients or next of kin)* was approximately 2, being occasionally experienced. Therefore, we suggest labeling this class* “occasionally exposed to aggression*.*”* In the fourth class, the average of these two items increased above 3. Hence, the respondent reported monthly aggression from patients or next of kin, leaving us with the label* “monthly exposed to aggression”* for this class. In the last class, labeled* “weekly exposed to aggression,”* the conditional average of aggression points to weekly reports of aggression from patients or next of kin. It is clear that the frequency of aggression was higher in this class. Almost 6% of the respondents reported weekly exposure to aggressive behaviors. In addition, 13% reported experiencing aggressive behaviors monthly. Almost 48% were occasionally exposed to aggression. Furthermore, 12% reported rare exposure to aggression, while only 21% reported no exposure to aggression at all (Table 2, first row). Hence, the first hypothesis was supported, as aggression from patients or next of kin was more commonly experienced by nurses than exposure to bullying behaviors.

### 4.3. Health-Related Outcomes of Aggression and Workplace Bullying

To compare the potential outcomes of aggression from patients or next of kin and exposure to bullying behaviors on physical and mental health-related quality of life, a MANOVA was used, adjusting for age and gender. Age was the only control variable with significant relations to the health-related outcome measures. There was a statistically significant difference between the different bullying classes on the combined dependent variables: *F*(6, 3784) = 11.85; *p* = 0.000; Wilk's Lambda = .96; partial eta squared = .02. When considered separately with ANOVAs, the only significant difference was found for the Mental Component Summary Score (MCS) (see Table 3). An inspection of the estimated marginal mean scores for MCS among the bullying classes, adjusted for age and gender, indicated that respondents in the* “frequently bullied”* class reported the lowest level of mental health-related quality of life (*M* = 39.47; SE = 1.70), as compared to the other classes.

Regarding aggression from patients or next of kin, there was no significant difference on the combined dependent variables:* F*(8, 3784) = 1.43;* p* = 0.18; Wilk's Lambda = .99; partial eta squared = .000, indicating no difference between the different aggression classes in relation to reported physical and mental health-related quality of life. Hence, the second hypothesis was supported, as exposure to bullying behaviors seems to have more severe health-related outcomes for those targeted than aggression from patients or next of kin.

Furthermore, the interaction effect between aggression from patients or next of kin and exposure to bullying behaviors on the combined dependent variables was not significant:* F*(24, 3784) = 1.02;* p* = 0.44; Wilk's Lambda = .99; partial eta squared = .01, indicating that the relationship between exposure to bullying behaviors and health-related quality of life was not dependent on the presence of aggression from patients or next of kin, or vice versa.

## 5. Discussion

The aim of the present study was to compare exposure to aggression from patients or next of kin and exposure to bullying behaviors in terms of prevalence and health-related quality of life outcomes. Furthermore, we wanted to determine if these stressors are part of a conglomerate experience or if the nurses' tendencies to report aggression and bullying differ and thereby being unrelated. Firstly, a two-latent-class-factor solution fitted better the data than a latent class cluster solution. Secondly, the correlation between the two factors was rather small. This indicates that these stressors were not intertwined phenomena constituting a conglomerate experience by the nurses. Furthermore, both aggression from patients or next of kin and exposure to bullying behaviors came in many levels of frequency with only a small minority of nurses experiencing systematic and frequent exposure. With respect to prevalence, aggression was by far the most common stressor and its reported frequency was also higher, supporting H1. However, only exposure to bullying behaviors was related to negative health-related outcomes in terms of reduced mental health-related quality of life, supporting H2. The latter finding strengthens previous research findings indicating that insider violence, in our case workplace bullying, is a major workplace distress factor which is more negatively related to health-related outcomes than is outsider aggression [7, 25].

### 5.1. Aggression Versus Workplace Bullying

In the present study, a two-factor model with five levels for each factor had the best fit for the data, with aggression from patients or next of kin being far more prevalent than exposure to bullying behaviors from colleagues. Nurses spend most of their working time with patients which may contribute to higher prevalence of aggression from patients in this occupation compared to other health professional groups [63]. Illustrative of this, the result in the present study corresponds with previous research comparing the prevalence of insider and outsider violence among nurses. In Farrell and colleagues' [64] study of Australian nurses, 60% reported being exposed to verbal abuse. Within this group, 74% reported verbal abuse from patients/clients, while 29% reported verbal abuse from colleagues. On the other hand, 24% of the total sample reported being exposed to physical abuse. Within this group, 97% had experienced physical abuse from patients/clients, while only 4% reported physical abuse from colleagues. Hence, outsider abuse was by far more prevalent than was insider abuse.

With regard to aggression from patients or next of kin, the results of the present study showed that 21% had a probability of reporting no aggression, while 6% were likely to report weekly exposure. Previous studies have shown that exposure to violence among nurses seems to vary between different European countries. In a study by Estryn-Behar and colleagues [3], nurses in Belgium (23%), Germany (28%), the UK (29%), and France (39%) reported the highest rates of frequent exposure (monthly or more), while nurses in Norway (9%) reported the lowest rates of violent episodes from patients and relatives.

With regard to exposure to bullying behaviors, the results in the present study showed that the probability of being part of the target class was 2%. On the other hand, the probability of being a nontarget was 50%. Parallel research has been done on the latent class cluster structure of NAQ in Great Britain [43] and Spain [65]. Despite differences in the numbers of clusters in these studies, the probability of not being a target of bullying was approximately the same (i.e., 30%), as well as the probability of being part of the respective severe bullying cluster (i.e., 5%). However, these studies showed slightly higher prevalence of bullying exposure than the present study did.

An explanation of the difference in prevalence between aggression and bullying may be the fact that patients more often behave in an aggressive manner against nurses as they are probably more likely to get away with it while venting anger and obtaining rewards in the process [5]. The target may also have less problems confronting and reporting these kinds of attacks as aggression from patients in many cases is seen as part of the profession [41] and therefore is not related to the nurse personally. Being bullied, on the other hand, is shown to be related to self-blame and shame responses in the nurses [66], and one can question whether these negative behaviors have been underreported in the present study. However, the lower prevalence of aggression and bullying at work in Norway, compared to other European countries, may also be explained by a more egalitarian and feminist culture which may indicate less tolerance for aggressive behaviors and power abuse in these workplaces (e.g., [67]). Also, a combination of cold climatic conditions and high national wealth may create a culture of cooperation with generally low levels of harassment [68]. Norwegian employees are furthermore protected by the Norwegian Work Environment Act, which bans harassment to occur in the workplace and protects the employees from inappropriate behaviors and stress [69]. Moreover, the Labor Inspectorate serves as a controlling authority, by giving advice, requiring investigation and the development of policies and procedures, and engaging in appropriate activity to solve the problem [70]. Hence, Norwegian employees may be in a position which protects them against high levels of violence at work, including both aggression and bullying, although it must be mentioned that the legal frameworks do not necessarily work in practice as intended.

### 5.2. Exposure to Bullying Behaviors and Health

Neither aggression from patients or next of kin nor exposure to bullying behaviors at work was related to nurses' perceived physical health-related quality of life. However, the results showed that being exposed to bullying behaviors was related to reduced mental health-related quality of life. These findings correspond with earlier research in a wide array of samples showing that, generally, bullying is a predictor of mental health problems over and above other work-related stressors (e.g., [71–75]).

Albeit the literature comparing health-related outcomes of bullying from colleagues and aggression from patients specifically is scarce, there are some studies comparing aggression from coworkers with aggression from patients, which to some degree parallels the result in the present study. In an Italian study among nursing students and nurses, the results showed that both physical violence and nonphysical violence from colleagues had a stronger relationship with psychological problems than patient- and relatives-initiated violence [2]. In LeBlanc and Kelloway's study [7], the results showed that public-initiated violence and aggression were differently associated with personal and organizational outcomes as compared to coworker-initiated aggression. The former was mainly associated with intent to leave, while the latter was negatively related to emotional well-being, psychosomatic well-being, and affective commitment. Illustrative of this, Merecz et al. [8] reported that aggression from colleagues was a stronger predictor of negative changes in health among nurses, despite verbal aggression from patients being the most common type of aggression. Also, in the meta-analysis by Hershcovis and Barling [6] on workplace aggression and various work and health outcomes, coworker aggression had a stronger adverse relationship with physical well-being than outsider aggression. There were, however, no significant differences between the groups in terms of depression, emotional exhaustion, and general health. Hence, even though patient-initiated violence increases the level of psychological stress and anxiety in nurses [76], it seems like systematic nurse-to-nurse aggression is perceived as the most distressing of the two [25], as indicated in the present study regarding workplace bullying.

An essential distinction between the two stressors included in this study concerns the duration of the problem related to a given perpetrator or perpetrators, where bullying is a process carried out over time by the same perpetrator(s), while incivility or aggression by patients typically is of a more sporadic character. A relevant theoretical explanation for this difference may be found in the CATS theory [36], where prolonged activation, first cognitive and then physiological, increases the risk of reduced health. Hence, one-time occurrences or short-time exposure may not cause ill-health to the same degree, especially when the target is able to cope with the situation, even if it tends to happen again with other perpetrators. Furthermore, in a hospital setting, patients usually stay for a short period of time, and the continuous replacement of patients may contribute to the evaluation of patients as part of an out-group. In a study by Riva and Andrighetto [77], social punishment (e.g., being humiliated in front of colleagues or being excluded from a group of friends) was perceived as less painful when the target was an out-group member as compared to an in-group member. Hence, it may be that nurses do distance themselves from the patients and that the aggressive behaviors are perceived as less harmful than bullying from colleagues of whom the nurses more highly identify themselves with and whom they may depend on at a daily basis. Also, the patients' illness may make their actions being perceived as less intentional. Illustrative of this, the results in a systematic review by Hahn and colleagues [1], among employees in general health care settings, indicated that the patients' health status, like dementia, recovering from unconsciousness, drug withdrawal, and mental illness, inclined them to be aggressive towards the staff. This illustrates the fact that nurses may believe that patients do not intend to hurt them or that the assault is part of the nature of nursing work [41]. Being exposed to bullying behaviors may, on the other hand, be evaluated as more insulting, because the nurses are not always in a position to withdraw from on-going interactions with colleagues and superiors [25] and thereby affect health negatively. When being targeted by members of the in-group, it may thus be harder to seek help, with impaired health as a possible outcome of prolonged worrying.

### 5.3. Methodological Considerations

This study has several strengths. First of all it uses latent class (LC) analysis to determine the relationship between aggression and workplace bullying, a method being very suited because it does not depend upon distributional assumptions [47, 48], which is often a problem in studies of bullying and aggression. Also, a MANOVA was conducted to determine health-related quality of life outcomes of aggression from patients or next of kin and exposure to bullying behaviors. Hence, we have likely avoided a type I error inflation because of a multiple testing procedure that would arise from repeating several ANOVAs. A strength is also the large sample size (*N* = 2059).

However, there are some limitations that should be mentioned. As most respondents are females (90.2%), it may weaken our ability to generalize the results to other more gender balanced occupations. Furthermore, use of cross-sectional data hinders the possibility to draw causal explanations for the findings. Also, the response rate was somewhat low (38.1%), indicating that there is a possibility for selection bias to occur. Hence, those with health problems and experiences with exposure to abuse at work could possibly have chosen not to participate in the study, and thereby the results may have been affected. This may also be related to the “healthy worker effect,” where healthy workers have a higher possibility of attending work than those who are sick [78]. When the data are based on self-reports, there is also an increased risk of common method variance [79]. However, the behavioral nature of the items measuring exposure to bullying behaviors and aggression from patients or next of kin should reduce this problem somewhat through their more “objective” nature. One other issue concerns the assessment of aggression from patients or next of kin, where the item* “difficult patients or next of kin”* may not correspond with aggressive patients or next of kin per se. The reliability of the scale was, however, acceptable (*α* = .81), and the scale is assumed to be a measure of patient aggression, although we acknowledge that better framing of this particular item could have been more appropriate.

## 6. Conclusion

To our knowledge, this study is one of the first to simultaneously compare the nature and health-related outcomes of patient aggression and workplace bullying in the nursing profession. The results suggest that aggressive behaviors from patients or next of kin and exposure to bullying behaviors are independent experiences and are seen as different constructs in the eyes of the beholder. Although aggression from patients or next of kin turned out to be more prevalent than exposure to bullying behaviors, the latter was the only one related to negative health-related outcomes among those targeted, in terms of reduced mental health-related quality of life. Hence, workplace bullying, although few are highly exposed, has a more severe impact on nurses' mental health than patient or next of kin aggression, at least in the present sample of Norwegian nurses. Information about the difference in how these stressors affect nurses' health functioning may increase managers' and nurses' understanding of the importance of reducing these stressors at work and in particular exposure to workplace bullying in order to maintain the nurses' good mental health functioning and well-being. Following this, the results in the present study suggest that intervention strategies should be implemented by health care management in order to generate and maintain a positive psychosocial work environment with zero-tolerance for bullying.

## Figures and Tables

**Table 1:** Models and their fit statistics.

	BIC (LL)	*N* par	*L* ^2^	PRE in %	Bootstrap *p* value *L*^2^	Class. err. (%)
Baseline model	30067.79	44	9608.64		0.000	0
1 cfa factor 5 classes	27483.56	60	6902.99	28.16	0.000	29.71
2 cfa factors 3, 3	26259.74	61	5671.57	40.97	0.006	19.37
2 cfa factors 4, 4	26065.31	63	5461.97	43.16	0.036	26.63
2 cfa factors 4, 5	26051.84	64	5440.91	43.37	0.056	26.68
2 cfa factors 5, 5	26033.25	65	5414.73	43.65	0.062	26.48
2 cfa factors 6, 5	26032.60	66	5406.49	43.73	0.024	38.37
**2 cfa factors 5, 5 errorcov 1 and 10**	**26016.70**	**66**	**5390.60**	**43.90**	**0.050**	**24.82**

*Note.* BIC: Bayesian Information Criterion; *N* par: number of parameters in the statistical model; *L*^2^: squared log-likelihood; PRE: proportion reduction of error; Class. err.: classification errors. Models are divided into a one-factor model, bifactorial models with different numbers of classes, and a final model with two factors including five classes each, where the error correlation between items 1 and 10 is .20. The model with the best fit is presented in bold.

**Table 2:** Conditional means of responding to the different classes of aggression from patients or next of kin and workplace bullying.

Factor (conditional means)	Latent classes and their size (%)									
	Exposure to bullying behaviors					Aggression from patients or next of kin				
	Never	Hardly	Rarely	Occasionally	Frequently	Never	Rarely	Occasionally	Monthly	Weekly
	50%	6%	29%	13%	2%	21%	12%	48%	13%	6%
*Exposure to bullying behaviors*										
(1) Someone withholding information which affects your performance	1.2648	1.4072	1.6189	1.9585	2.5274	1.3367	1.4026	1.4935	1.6635	1.8668
(2) Repeated reminders of your errors or mistakes	1.0462	1.1164	1.2731	1.5803	2.2091	1.1250	1.1660	1.2211	1.2889	1.3651
(3) Persistent criticism of your work or work-effort	1.0051	1.0234	1.1025	1.3994	2.3578	1.0512	1.0798	1.1217	1.1780	1.2475
(4) Spreading of gossip and rumors about you	1.0022	1.0165	1.1145	1.5315	2.4398	1.0581	1.0931	1.1438	1.2113	1.2931
(5) Having insulting or offensive remarks made about your person, attitudes, or your private life	1.0031	1.0199	1.1173	1.4871	2.2548	1.0562	1.0886	1.1350	1.1960	1.2692
(6) Being ignored or excluded	1.0210	1.0579	1.1521	1.3625	1.7819	1.0685	1.0939	1.1284	1.1715	1.2205
(7) Being ignored or facing a hostile reaction when you approach	1.0101	1.0408	1.1540	1.4877	2.3371	1.0695	1.1040	1.1528	1.2163	1.2917
(8) Practical jokes carried out by people you do not get along with	1.0078	1.0328	1.1296	1.4245	2.1261	1.0586	1.0882	1.1302	1.1846	1.2492
(9) Being shouted at or being the target of spontaneous anger	1.0350	1.0924	1.2317	1.5273	2.0532	1.1048	1.1414	1.1908	1.2517	1.3201
*Aggression from patients or next of kin*										
(10) Difficult patients or next of kin	2.1623	2.3269	2.5111	2.7189	2.9575	1.5260	1.9052	2.3203	3.3679	4.3380
(11) Verbal assaults or spontaneous tantrums from patients or next of kin	1.6811	1.8670	2.0693	2.2849	2.5109	1.000	1.0104	1.9193	3.0292	4.2279
(12) Physical assaults or threats of such assaults from patients or next of kin	1.2181	1.2941	1.3890	1.5043	1.6395	1.0107	1.0476	1.1997	1.7210	3.0227

*Note.* Exposure to bullying behaviors is measured by the Negative Acts Questionnaire (NAQ).

**Table 3:** Bivariate effects of aggression from patients or next of kin and workplace bullying on health-related quality of life outcomes, from the MANOVA output.

Variables		*F*	df	*p*	Partial *n*^2^
Age	PCS	70.21	1	0.000	.036
	MCS	5.89	1	0.015	.003
Gender	PCS	0.16	1	0.691	.000
	MCS	0.23	1	0.634	.000
Aggression from patients or next of kin	PCS	0.30	3	0.876	.001
	MCS	2.24	3	0.063	.005
Workplace bullying	PCS	1.31	4	0.268	.002
	MCS	19.81	4	0.000	.030
Aggression from patients or next of kin *∗* workplace bullying	PCS	0.59	12	0.854	.004
	MCS	1.32	12	0.201	.008

*Note.* PCS: Physical Component Summary Score; MCS: Mental Component Summary Score.
